# Harnessing *Schistosoma*-associated metabolite changes in the human host to identify biomarkers of infection and morbidity: Where are we and what should we do next?

**DOI:** 10.1371/journal.pntd.0012009

**Published:** 2024-03-21

**Authors:** Mireille Kameni, Fungai Musaigwa, Leonel Meyo Kamguia, Severin Donald Kamdem, Gladice Mbanya, Poppy H. L. Lamberton, Justin Komguep Nono

**Affiliations:** 1 Unit of Immunobiology and Helminth Infections, Laboratory of Molecular Biology and Biotechnology, Institute of Medical Research and Medicinal Plant Studies (IMPM), Ministry of Scientific Research and Innovation, Yaoundé, Cameroon; 2 Department of Microbiology and Parasitology, University of Bamenda, Bambili, North-West Region, Cameroon; 3 Department of Pathobiology, School of Veterinary Medicine, University of Pennsylvania, Philadelphia, Pennsylvania, United States of America; 4 Division of Microbiology and Immunology, Department of Pathology, University of Utah School of Medicine, Salt Lake City, Utah, United States of America; 5 School of Biodiversity, One Health & Veterinary Medicine, University of Glasgow, Glasgow, United Kingdom; 6 Wellcome Centre for Integrative Parasitology, University of Glasgow, Glasgow, United Kingdom; 7 Division of Immunology, Health Science Faculty, University of Cape Town, Cape Town, South Africa; Uniformed Services University: Uniformed Services University of the Health Sciences, UNITED STATES

## Abstract

Schistosomiasis is the second most widespread parasitic disease affecting humans. A key component of today’s infection control measures is the diagnosis and monitoring of infection, informing individual- and community-level treatment. However, newly acquired infections and/or low parasite burden are still difficult to diagnose reliably. Furthermore, even though the pathological consequence of schistosome egg sequestration in host tissues is well described, the evidence linking egg burden to morbidity is increasingly challenged, making it inadequate for pathology monitoring. In the last decades, omics-based instruments and methods have been developed, adjusted, and applied in parasitic research. In particular, the profiling of the most reliable determinants of phenotypes, metabolites by metabolomics, emerged as a powerful boost in the understanding of basic interactions within the human host during infection. As such, the fine detection of host metabolites produced upon exposure to parasites such as *Schistosoma* spp. and the ensuing progression of the disease are believed to enable the identification of *Schistosoma* spp. potential biomarkers of infection and associated pathology. However, attempts to provide such a comprehensive understanding of the alterations of the human metabolome during schistosomiasis are rare, limited in their design when performed, and mostly inconclusive. In this review, we aimed to briefly summarize the most robust advances in knowledge on the changes in host metabolic profile during *Schistosoma* infections and provide recommendations for approaches to optimize the identification of metabolomic signatures of human schistosomiasis.

## Introduction

Schistosomiasis, also known as bilharzia, is a debilitating and potentially fatal neglected tropical disease (NTD) caused by some species of blood trematode parasites of the genus *Schistosoma*. Schistosomiasis infection in humans is mainly caused by *S*. *mansoni*, *S*. *haematobium*, or *S*. *japonicum* [1–3], which causes noticeably morbid and life-threatening diseases mostly in children with symptoms ranging from impaired growth, reduced learning abilities, fever, anemia, abdominal pain, organ enlargement, reduced vaccine response [4], and tissue damage [5]. As the second most debilitating parasitic disease in the world after malaria, 700 million people are living in endemic areas and approximately 250 million people are infected worldwide with more than 200,000 deaths annually [2]. An estimated 85% of global *Schistosoma* infections occur in Africa, highlighting a public health concern in resource-constrained communities [1]. In these areas, schistosomiasis is primarily propagated by poverty (especially inadequate sanitation) correlated with the absence of safe water, with daily household chores such as washing and cooking, and recreational activities including swimming occurring in unprotected and contaminated freshwater sources [6]. Additionally, water-related economic activities such as contaminated freshwater irrigation and fishing can also spread the parasite to people, making its control difficult in regions where such activities are essential for the survival of most households [1].

## The infection cycle and associated pathophysiology

The infection is contracted by humans and animals when coming into contact with infested water, containing infective larvae of *Schistosoma* spp. released from freshwater snails (the intermediate hosts). Upon contact, the infective larvae of *Schistosoma* spp, cercariae, penetrate the host skin. After invading the host skin, cercariae move towards the dermal veins [2], and at this point, larvae metamorphose and develop into schistosomula with strong coats that block the host’s immune response [7]. The schistosomula enter the systemic circulation via the venous blood vessels and are transported to the lungs and pulmonary capillaries to join the arterial circulation. As a result of schistosome cercariae infection, the human host may exhibit dermatitis caused by host responses to cercariae penetration of the skin and bronchopulmonary distress as a result of schistosomula passing through the lungs [2,3].

Once the schistosomula arrive in the hepatic portal system, they migrate to the mesenteric veins of the liver or the uro-genital vasculature where they then mature into adults. More pronounced symptoms will occur with parasite development and increased antigenic release which may include fever, dry cough, weakness, headache, and abdominal symptoms that are not pathognomonic of schistosomiasis alone but are usually reported during the acute phase of the disease, also called Katayama fever or Katayama syndrome [2]. Acute schistosomiasis is typically seen in nonimmune travelers, rather than residents of endemic areas. In rare cases, acute schistosomiasis may cause severe symptomatology, including cardiac and neurological complications, depending on the size of the infective inoculum (cercaria burden) and the intensity of the host’s immune response to the parasite antigens [8,9].

In this hepatic/uro-genital vascular network, adult schistosomes now fully developed, will mate and produce highly antigenic eggs that are inefficiently expelled via the intestine or bladder wall, and excreted from the body in the urine or feces. This results in a considerable amount of eggs being sequestered in tissues such as the liver, gastrointestinal tract, or urogenital compartment depending on the *Schistosoma* spp. [7]. The egg deposition from adult worms that will lodge in host tissues will now cause the critical part of schistosomiasis morbidity. In this phase, which is termed chronic schistosomiasis, hundreds to thousands of eggs are laid each day by adult worms living in the mesenteric branches of the portal vein along the intestinal wall (*S*. *japonicum* and *S*. *mansoni* infection) or the venous plexus around the urinary bladder (*S*. *haematobium* infection). The blood carries a large number of the eggs to the liver (*S*. *mansoni*) and other organs (bladder or genitalia for *S*. *haematobium*), where they are trapped and the continuous deposition of eggs cause a granulomatous response resulting in chronic inflammation lesions [2,3,7]. Under the sustained, untamed, and overwhelming action of the host immune response to the sequestered parasite eggs and excreted antigens, the ova-surrounding granulomatous structures are gradually replaced by fibrotic meshes that progressively and deleteriously replace the tissue parenchyma as the disease is left untreated and the chronicity is consolidated [7]. These lesions and subsequent complications translate into the pathognomonic clinical features of the disease including uro-genital bleeding and haematuria (*S*. *haematobium*), hepatosplenomegaly, pathological liver fibrosis, and portal hypertension (*S*. *mansoni* and *S*. *japonicum*) [10] which can be fatal if left untreated.

The species-specific sequestration of *Schistosoma* spp. eggs is consequent to the species-specific mode of egg excretion. Notably, *S*. *mansoni* and *S*. *japonicum* (species causing intestinal schistosomiasis) eggs are excreted in human feces, while eggs from *S*. *haematobium* (causing urogenital schistosomiasis) are excreted in the urine [1–3,7]. Exceptionally, schistosome eggs or adult worms may also take up residence in unusual tissues, giving rise to ectopic forms of schistosomiasis which are often difficult to clinically diagnose and can lead to aberrant morbidities [8].

## Control efforts against schistosomiasis

**Fig 1:**
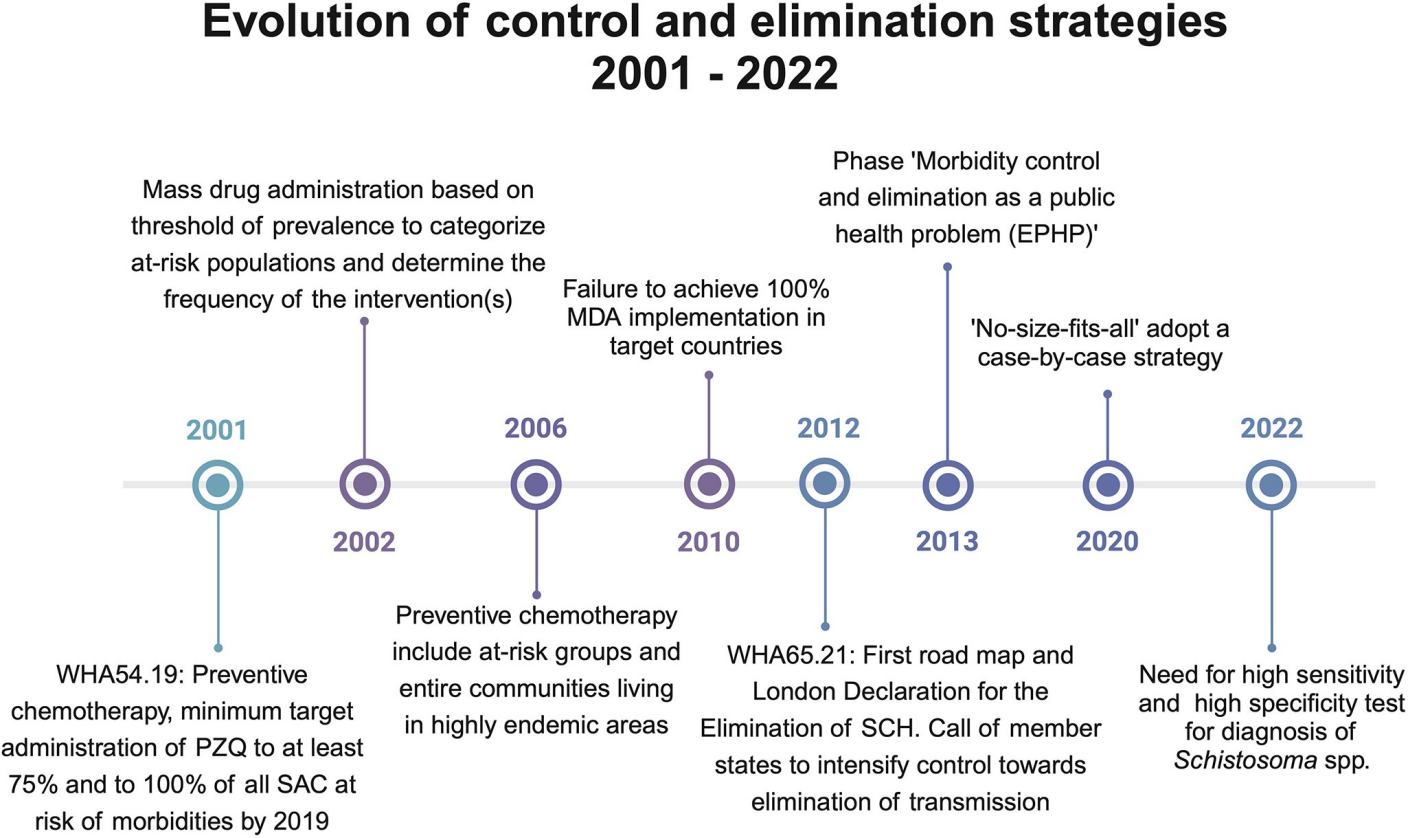
Timeline of global schistosomiasis control and elimination strategies (2001–present). Created in Biorender.com.

Throughout history, global strategies for controlling schistosomiasis have focused on preventing and/or treating the infection and disease through mass drug administration (MDA), with some countries implementing early and reliable diagnosis of infections and robust monitoring of the disease progression to ensure effectiveness of the actions [11]. Since their launch in multiple countries in the 1950s, schistosomiasis control programs have primarily focused on reducing disease burden in endemic areas by reducing snail population, removing vegetation, and treating all *Schistosoma* spp. with oxamniquine (for *S*. *mansoni*) and more recently with praziquantel (PZQ), active against all *Schistosoma* spp. [12]. The fight against schistosomiasis gained further impetus in 2003 when national control programs across sub-Saharan Africa were scaled up, supported by PZQ drug donations and new World Health Organization (WHO) recommendations and guidelines [13]. In these larger control programs, the aim was to expand the area subjected to MDA of PZQ and reduce infection intensities, as well as morbidity [14] and to measure (by 1 urine filtration or Kato-Katz) the prevalence of infection in the most vulnerable community group, the school-aged children. Moreover, stakeholders took measures beyond the WHO 2013 report and recommendation to decrease *Schistosoma* infection’s prevalence and intensity, primarily by strategically and periodically administering PZQ to high-risk adults in endemic areas, as well as to school-age populations in endemic areas [9,12]. In order to achieve such a goal, *Schistosoma* infection needs to be routinely diagnosed and the burden of infection determined as a linear proxy of morbidity. As such, control of morbidity was classified as achieved when the prevalence of heavy infections fell below 5% [9,12]. The use of egg burden as a metric for assessing MDA effectiveness has remained common practice, despite the limitations of microscopical-based Kato-Katz and urine filtration diagnostic techniques. Subsequently, the 2030 resolution NTDs, aims for the elimination of schistosomiasis as a public health problem [12,15,16]. This elimination as a public health problem is currently defined as achieving a target prevalence of less than 1% heavy infections among schoolchildren, as defined by a Kato-Katz-based microscopical assessment of patients’ excreta for *S*. *mansoni* [12,15,16]. Consistently through these evolving global schistosomiasis strategies [12–14], large-scale treatment through MDA of PZQ has proved to be effective and powerful in decreasing prevalence by 60% among school-aged children over the last 20 years [17]. However, the community remains cognizant of the need to diagnose infection early on and to grade morbidity reliably with easy-to-use tools. This need for new infection and morbidity markers for improved diagnostic and morbidity monitoring tools was formally raised in the most recent road map for the elimination of schistosomiasis as a public health problem by 2030 and the accompanying guidelines published in 2022 (Fig 1 [16]).

## Current diagnostic tools for *Schistosoma* infection and limitations: Why is there a need for alternative/complementary diagnostic markers and tools?

Effective diagnostics are a prerequisite to reaching the goals set in the 2030 road map for NTDs [18]. Robust diagnostic tools are key components of the strategy to reduce morbidity and costs of schistosomiasis disease control programs, e.g., from confirmation of infection and disease to mapping, screening, surveillance, monitoring, and evaluation. Combined with clinical observations profiles (hepatosplenomegaly, bloody stool or diarrhea for intestinal schistosomiasis and hematuria for urogenital schistosomiasis), multiple and effective diagnostic techniques are available for the identification of *Schistosoma* (Table 1). Examples of these are the microscopy detection of parasite eggs in the patient’s excreta, the immunological detection of parasite antigens in their body fluids, or the molecular detection of parasite nucleic acids in their tissues. However, they often have important drawbacks that limit their use as efficient large-scale control tools [19,20]. Specifically, the most commonly used, and WHO endorsed, diagnosis of *Schistosoma* infections are microscopic identification of eggs in the feces of clinical specimens via the Kato-Katz thick smear technique for stool (*S*. *mansoni*) or in the urine following the urine filtration method (*S*. *haematobium*). Despite their specificities, cost-effectiveness and ease of implementation, microscopy methods often fail to detect low-intensity infections, predominantly found in low-endemicity areas or post treated with PZQ [21,22]. Antibody-based serological tests can also be used; however, these are critically limited by the presence of antibodies in previously infected but cured individuals, a drawback that has also be reported for the DNA-based diagnostic by PCR [19,20]. Although highly specific to the parasite, the molecular detection of RNA is very poorly sensitive and strongly depends on the ability to obtain patients’ biopsies with transcriptionally active parasite material, which is not easily implementable in the field. Conversely, in addition to microscopical-based diagnostic tools, WHO recommends the use of a point-of-care (POC) lateral flow test based on circulating cathodic antigen (CCA) detection [23], where CCA identification reflects the presence of viable worms in current infections. This is a successful implementation of a biomarker of infection and its reliable use for *S*. *mansoni* diagnosis [23]. Although easy and quick to perform and interpret, this method is insensitive to *S*. *haematobium*, and validation has not been demonstrated for some other species [24]. In addition, POC-CCA has also been associated with potential high rates of false-positive results, and inconclusive results caused by “trace” readings. Compared to the increasingly validated up-converting particle lateral flow circulating anodic antigen test (UC-LF CAA), POC-CCA appears to be underperforming [25,26]. Hence, POC-CCA might unreliably estimate disease burdens as well and consequently limit cost-efficient strategic control interventions. At present the UCP-LF-CAA test requires a laboratory and is more expensive that the POC-CCA, but it is suitable for all human infecting *Schistosoma* spp. The UCP-LF-CAA test is currently under development as a rapid diagnostic test to assist the currently limited diagnostic tools in the fight against schistosomiasis, but a sensitive and specific field deployable CAA test has yet to be commercially produced. The need for improved markers in the context of *Schistosoma* infection for monitoring and surveillance is increasingly reinstated by the WHO in the ultimate quest for the most refined and deployable diagnostic test or combination of tests for the unequivocal surveillance of the true prevalence of active infection whatever the stage of advancement of the disease [15,16]. In addition, improved diagnostics would aid in the formation of longer term wide-scale monitoring of control program success, and the identification of hotspots to inform strategic program implementation, running time and improvements to programs where required [19,20]. To this end, the WHO has recently published a target product profile for new diagnostics to detect *Schistosoma* infections, for 2 end case scenarios [27].

**Table 1:** Current and foreseeable *Schistosoma* diagnostic strategies: Benefits and limitations.

	Current diagnostic methods	Benefits	Limitations	Suggestions for improvement
Current diagnostic assays	**Microscopic examination of stool or urine samples** (e.g.: Kato-Katz, Urine filtration)			- Explore novel microscopy techniques or staining methods to improve sensitivity in low-intensity infections. - Explore the use of automated microscopy systems or digital imaging technologies to increase the efficiency and accuracy of microscopic examination. - Develop automated image analysis algorithms for the rapid and accurate quantification of parasite eggs in microscopic samples.
	- Direct visualization of parasite eggs	x		
	- Low cost and widely available	x		
	- Established and well-known method	x		
	- Limited sensitivity in low-intensity infections		x	
	- Requires skilled technicians for accurate interpretation		x	
	- Time-consuming and labor-intensive		x	
	**Nucleic acid amplification** (e.g.: classical PCR, nested PCR, multiplex PCR, real-time quantitative PCR, molecular barcoding, and loop-mediated amplification (LAMP))			- Develop cost-effective PCR alternatives. - Simplify PCR protocols without compromising sensitivity and specificity.
	- High sensitivity and specificity	x		
	- Can detect low-intensity infections	x		
	- Enables species identification	x		
	- Requires well-equipped laboratory facilities		x	
	- Costlier compared to microscopy		x	
	- Requires skilled personnel for reliable results		x	
	**Antigen detection assays** (e.g.: circulating cathodic antigen (CCA) in urine, circulating anodic antigen (CAA))			- Enhance the specificity of antigen detection assays through improved antigen selection and validation processes. - Invest in the development of multiplex assays that can simultaneously detect and differentiate multiple schistosome species, enhancing the diagnostic specificity. - Explore the use of novel biomarkers or antigens specific to different stages of schistosome infection to improve the sensitivity and specificity of antigen detection assays.
	- Rapid results	x		
	- Suitable for field settings	x		
	- High sensitivity in active infections	x		
	- May yield false positives		x	
	- Limited specificity in endemic areas		x	
	**Serological tests**			- Conduct further research to identify specific antibody markers and reduce cross-reactivity in serological tests. - Investigate alternative sample types, such as saliva or dried blood spots, that could facilitate noninvasive or easier sample collection for diagnostic purposes.
	- Detects antibodies produced in response to infection	x		
	- Useful in areas with low transmission	x		
	- Can provide information on past exposure	x		
	- False positives due to cross-reactivity		x	
	- Limited sensitivity in acute infections		x	
	**Point-of-care tests (POCTs)**			- Develop of more sensitive and affordable point-of-care tests (POCTs) for the detection of low-intensity infections. - Conduct comparative studies to evaluate the performance and reliability of different POCTs under diverse field conditions. - Conduct cost-effectiveness analyses to evaluate the economic impact and feasibility of implementing different diagnostic methods in various healthcare systems.
	- Rapid and convenient	x		
	- Can be used in resource-limited settings	x		
	- Suitable for mass screening	x		
	- Lower sensitivity compared to PCR		x	
	- Limitations in detecting low-intensity infections		x	
	- Variable performance across different POCTs		x	
**Diagnostic methods based on future orientations**	**Omics-based (transcriptomics-proteomics-metabolomics)**			- Develop simplified and cost-effective OMICS platforms that can be implemented in resource-limited settings. - Establish centralized databases of biomarkers to support OMICS data interpretation and analysis.
	- Enables simultaneous analysis of multiple samples and large datasets	x		
	- Provides high-resolution information	x		
	- Offers potential for pathogen identification and monitoring of pathogen’s as well as hosts’ specifics	x		
	- Requires sophisticated infrastructure and bioinformatics expertise		x	
	- Costly, limiting accessibility in resource-limited settings		x	
	- Data analysis and interpretation can be complex and time-consuming		x	
	**Microarrays**			- Standardize microarray platforms and probe libraries for broader application and compatibility across diagnostic laboratories.
	- Allows for parallel detection of multiple targets	x		
	- High-throughput screening capabilities for genotyping or gene expression analysis	x		
	- Potential for multiplexing and high sensitivity	x		
	- Requires specific probes for target detection and may lack flexibility		x	
	- Relatively expensive, limiting widespread adoption		x	
	- Interpretation and data analysis can be challenging without appropriate expertise		x	
	**Machine learning (ML) algorithms**			- Invest in the development of user-friendly ML algorithms and tools tailored for medical professionals to enhance accessibility and usability. - Promote the creation of open-access, well-curated datasets for training ML and AI models, ensuring diversity and representation. - Conduct rigorous validation studies to assess the clinical utility, accuracy, and impact of ML and AI systems in real-world diagnostic settings. - Provide comprehensive training and education programs to empower healthcare professionals with the necessary skills to effectively utilize high-throughput technologies, machine learning, and AI in clinical practice.
	- Enables pattern recognition and classification of complex data	x		
	- Can handle large datasets and extract meaningful features	x		
	- Potential for improved accuracy and efficiency in diagnosis	x		
	- Dependence on high-quality, annotated datasets for training		x	
	- May require significant computational resources and expertise		x	
	- Interpretability and transparency of ML models may be challenging		x	
	**Artificial intelligence (AI) systems**			- Establish partnerships with local healthcare providers and NGOs to facilitate capacity building and training programs on the use of high-throughput technologies, machine learning, and AI in low-resource settings. - Establish collaborations between academic institutions, industry, and local healthcare providers to facilitate technology transfer, knowledge exchange, and sustainable implementation of high-throughput diagnostic solutions. - Conduct robust cost-effectiveness analyses to demonstrate the value and impact of implementing high-throughput technologies, machine learning, and AI in poor settings, thereby facilitating advocacy and resource allocation.
	- Capable of learning from diverse data sources and improving over time	x		
	- Potential for automated decision-making and real-time analysis	x		
	- Can integrate multiple data types for enhanced diagnostic performance	x		
	- Ethical and privacy concerns related to data usage and patient confidentiality		X	
	- Dependence on high-quality, curated datasets for training and validation		X	
	- Interpretability and transparency of AI algorithms require attention		X	

## Current tools to monitor schistosomiasis-related morbidity and limitations: Why the need for alternative/complementary morbidity markers and monitoring tools?

The morbidity associated with schistosomiasis is pernicious and insidious, primarily arising from the host’s untoward reaction against the sequestered parasite eggs [2,3,7]. The general assumption hitherto was that of a linearity between the egg burden and the severity of the pathology [5,28,29]. Such an assumed linearity has been at the basis of morbidity assessment proceedings for control programs whereby thresholds of disease burden were defined based on the prevalence of heavy infections, assumed to be indicative of severe pathology [12,15]. This has had some merit, streamlining the assessment of an apparent morbidity profile in treated communities easily without having to roll out expensive, logistically demanding and time-consuming ultrasound devices to unequivocally assess the degree of organ pathology in schistosomiasis patients. However, it is now becoming increasingly clear that a higher egg burden does not automatically translate into severe morbidity or that low burden infections are not prone to severe pathologies [30–32]. The relationship between intensity (as measured by excreted egg counts) and host morbidity at that time point has therefore become questionable, especially for *S*. *mansoni* [30–32]. In addition, the gold standard morbidity monitoring tool for tissue pathology, ultrasonography, poses a clear logistical problem, given its poor amenability to large-scale deployment in remote and/or poorer areas, where the disease is often most prevalent. It is under these premises that the need for morbidity markers to reliably monitor the progression of schistosomiasis morbidity, and its successful regression, becomes paramount en route to truly achieving the 2030 WHO goal of eliminating the disease as a public health problem.

## The relevance of host metabolomics in the identification of biomarkers of human diseases

Perturbation of the host homeostasis is typical of most infections and a robust basis for the biological marking of the infection and its disease, relative to the alterations reported in the host. Regardless of the alterations investigated at the gene or transcript level, proteins and metabolites are the most proximal and best sources of biomarkers [33]. This supports the widely emerging use of metabolomics. Metabolite analysis is used to identify novel biomarkers of infection and disease, which may be used to rationally design diagnostic, prophylactic, or therapeutic tools [33]. Recent advances in liquid chromatography coupled with tandem mass spectrometry (LC-MS/MS) have contributed to the rapid growth of this field, providing a snapshot of the physiological state of a biological system by profiling its small molecule metabolic products at a specific time and condition [34]. Profiling all metabolites and relating them to metabolic pathways is useful for the robust identification of key checkpoints associated with a given phenotype of the system [33,34]. Many cancers and other diseases have been studied using metabolomics to better understand the disease, aid in the detection of the disease, and/or aid in the development of treatment options [35–37]. Based on these checkpoints, diagnostic or therapeutic tools can be developed for the patient’s phenotype.

Nowadays, significant advances in spectroscopy-based metabolomics techniques favor flexible analyses and controlled diagnostics in a limited time compared to before [38]. The current trend towards automation and standardization of protocols regarding experimental design, sample handling, and pretreatment in metabolomic workflows is an effective way to improve clinical diagnosis, early detection, treatment prediction, and monitoring of treatment effectiveness [39]. Metabolomic studies on body fluids in diseased states [35–37] including helminth infections [38], shed light on some valuable biomarkers of several infectious diseases (Table 2). Due to the high sensitivity of metabolomics, it can often detect subtle biological changes. However, although altered metabolic activities due to parasitic infections have been studied in animal models [40,41], confirmation in humans is documented only in rare pilot studies [42,43]. The difficulty in validating such candidate biomarker often lies in the need for targeted and strictly designed follow up experiments. Challenges in human studies arise due to the effect of individual host heterogeneity on the variation of the metabolome whereby age, genetics, diet, and other lifestyle factors all create variation in the metabolome [44,45]. Case-control studies or longitudinal follow-up of the same individuals, reducing confounding factors, are often the basis for robust biomarker validation experiments but human studies are scarce to date. These limitations do not remove the powerful value of metabolomics in infectious disease research where the robust identification of small molecules, e.g., metabolites produced during infection, may elucidate potential diagnostic markers directly related to infection and associated pathology [33,38,39]. The use of this technique has recently gained momentum in schistosomiasis research but the sample sizes are small and the number of studies is limited (Table 2). Therefore, an appraisal of the current status of host metabolomics during schistosomiasis and a road map for future steps to be taken in achieving a robust mapping of the host metabolite biomarkers of infection and pathology are indicated and timely.

**Table 2:** Metabolomic profiles of mammalian hosts infected by *Schistosoma* spp.

Species	Stage of the disease	Biomarkers identified	Body fluids used	model	Tendency	References
*Schistosoma mansoni*	(in Children)	1) Dimethylamine 2) Hippurate 3) PAG 4) Trimethylamine 5) 2-Oxo glutarate 6) Acetate 7) Citrate 8) Fumarate 9) Pyruvate 10) Succinate 11) 2-Oxo isocaproate 12) 3-Hydroxy butyrate 13) Acetone 14) Creatine 15) Guanidino acetate 16) Methylguanidine 17) Formate 18) TMAO 19) Trans Aconitate 20) Trigonelline	Urine	Human (children, adults)	1) Down 2) Down 3) Up 4) Up 5) Down 6) Up 7) Up 8) - 9) Down 10)– 11) Down 12) Down 13) Down 14) - 15) Up 16) Up 17) - 18) Up 19) - 20) Down	[46]
		1) Tryptophan 2) Creatine 3) Benzoic acid 4) Uric acid 5) Glycolic acid	Urine	Mice	Up	[41]
		6) Hippuric acid 7) Citric acid			Down	
	Mild PPF (liver fibrosis)	1) Valine 2) Carbohydrates	Serum	Human		[47]
	Significant PPF	1) Alanine 2) N-acetylglucosamine 3) Glycolaldehyde				
*Schistosoma mekongi*	Early stage	1) Heptadecanoyl ethanolamide (saturated fatty acid) 2) Picrotin (GABA receptor stimulant) 3) Theophylline (caffeine metabolism)	Serum	Mouse	1-down 2-down 3-up	[48]
*Schistosoma haematobium*	Infection only	1) Adrenochrome O-quinone 2) 3-Succinoylpyridine	Plasma	Human	1-up 2-up	[43]
	Advanced (with bladder pathologies)	1) Modified estradiol/testosterone 2) N-Glycoloylganglioside GM2 3) Phosphatidylcholine (PC) 4) Phosphatidylethanolamine (PE) 5) LysoPC(14:0)/1HGPE	Plasma		1-down 2-up 3-up 4-down 5-up	
	Advanced + Infection	1) Indolylacryloylglycine (Tryptophan metabolism)	Urine		1-up	
*Schistosoma haematobium*	Early infection	1) Adenosine diphosphate 2) 3-phosphoglyceric acid 3) Adenosine monophosphate 4) Inosine 5) Asparagine 6) 2-hydroxybutyric acid 7) Sarcosine 8) Guanosine monophosphate 9) Glucose-6-phosphate 10) Ethanolamine phosphate	Serum	Human	Up	[42]
		1) Lactic acid 2) Choline 3) Serine 4) cis-asconitic acid 5) Histidine 6) and glutamic acid 7) ADP 8) 3-PG 9) AMP			Down	
*Schistosoma japonicum*	BALB/c mice	1) Dimethylhexane/methyloctane/ethylheptane 2) LysoPC(22:5) 3) L-Glutamic acid n-butyl ester 4) L-threo-3-Phenylserine 5) PC(22:6/0:0)/LysoPC(22:6) 6) PC(18:2/0:0)/LysoPC(18:2) 7) PC(19:0/0:0) 8) LysoPC(20:3) 9) PC(18:0/0:0) 10) LysoPE(18:2/0:0)/LysoPE(0:0/18:2)/PE(18:2/0:0) 11) PC(20:5/0:0) 12) L-Phenylalanine 13) Hexadecenyl acetate 14) Zizyphine A 15) PC(16:1/0:0) 16) Phthalic acid Mono-2-ethylhexyl Ester 17) PC(14:0/O-1:0) 18) PC(O-16:0/0:0) 19) 3-Hydroxy-2H-pyran-2-one 20) PC(O-16:0/2:0)/PC(0:0/18:0)/LysoPC(18:0) 21) PC(0:0/16:0)/PC(16:0/0:0)/LysoPC(16:0) 22) PC(20:2/0:0) 23) 5-Hydroxy-2,4-dioxopentanoate 24) PC(0:0/18:1) 25) LysoPC(18:2)/PC(18:2/0:0) 26) Dodecyl 2-methylpropanoate 27) LysoPC(20:4)/PC(20:4/0:0) 28) LysoPC(17:0) 29) PE(18:1/19:1)/PC(14:0/20:2)/PE-NMe(18:1/18:1	Serum	Mouse		[49]
	Severe combined immunodeficient (SCID) mice	1) Phytosphingosine 2) PE(22:6/0:0) 3) LysoPE(0:0/16:0)/PC(13:0/0:0) 4) 3-Hydroxy-10′-apo-b,y-carotenalb/Docosanedioic acid/Octyl hexanedioate 5) 2-Aminooctanoic acid 6) Glycerophosphocholine/sn-glycero-3-Phosphocholine 7) 8,11,14-Docosatriynoic acid/Neogrifolin/Neogrifolin/7alphaMethyl-4-pregnene-3,20-dione 8) 1,2,3,4,4a,9,10,10a-Octahydro-6-hydroxy-7-isopropyl-1,4adimethyl-1-phenanthrenemethanol 9) 7,13-Eicosadiynoic acid 10) 17alpha-Methyl-17beta-hydroxyandrosta-4,6-dien-3-one/4-Oxoretinol/8,11,14,18-Eicosatetraynoic acid 11) Palmitic amide 12) 3′-Sialyl-3-fucosyllactose 13) 9R-HETE/(+)-Beyerol 14) Eicosatetraenoic acid 15) Stearamide				
*Schistosoma japonicum*	Early infection (3days PI)	1) Phosphatidylcholine (PC) (22:6/18:0) 2) Colfosceril palmitate	Serum	Mouse	1-down 2-down	[50]
		1) Xanthurenic acid 2) Naphthalenesulfonic acid 3) Pimelylcarnitine	Urine		1-down 2-up 3-up	
	Late infection	*Glycerophospholipids* PC Phosphatidylethanolamine (PE) Phosphatidylserine Phosphatidylinositol LysoPC *sphingomyelin (SM) species* Ceramide (Cer) *carnitine species* Acetylcarnitine Oleoylcarnitin			down down up up	
*Schistosoma japonicum*	All stages of infection	1) Cerium 2) Glycerol tribenzoate 3) Catechin 7-glucoside 4) Uridine 5) Selenomethionine 6) Muramic acid 7) Allopurinol 8) Glyceric acid	Serum	Mouse	1) down 2) up 3) down 4) down 5) down 6) up 7) up 8) up	[51]
		9) 1-Methylinosine 10) Anigorootin 11) Dimethyl D-malate 12) PS(21:0/0:0) 13) N-Acetyl-d-glucosamine 14) PGE3 15) Deoxycholic acid 3-glucuronide			9) up 10) up 11) up 12) up 13) up 14) up 15) up	
*Schistosoma japonicum*	Late infection (chronic and advanced)	1) Ng,ng-dimethyl-l-arginine 2) Fenamiphos 3) L-saccharopine 4) 2-pyrrolidinone, 1-methyl- 5) Tyramine 6) Taurodeoxycholic acid 7) 3,4,5-trimethoxycinnamic acid	Intestinal (feces)	Human	1) up 2) up 3) up 4) up 5) up 6) up 7) down	[52]

## Host metabolomics during schistosomiasis: Status and outlook

*Schistosoma* infection and associated disease cause distinct alterations of the host metabolite profiles [42,43,48]. The metabolomic profile of schistosomiasis infection has been extensively explored in animal models [33,38–41,48] and recently reviewed [53]. The variety of metabolite pathways identified has enabled hypotheses to be explored and an increased understanding of the progression of the disease [54]. However, most of the metabolomics studies identified in this recent scoping review were animal-based, supporting the need for more human studies to identify and validate potential host metabolite biomarkers. The progress made through these initial studies provides strong starting points for shaping future robust metabolomics-based biomarking studies for human schistosomiasis.

In a seminal study on host metabolomics during schistosomiasis, Wang and colleagues induced chronic schistosomiasis in mice and used 1H nuclear magnetic resonance (NMR) spectroscopy to describe the metabolic signature of *S*. *mansoni* infection [55]. In this study, for the first time, the potential of metabolomics in deciphering parasitic infection with *S*. *mansoni* was demonstrated. Novel biomarkers that could potentially serve as a basis for future diagnostic tests due to their high sensitivity and specificity compared to the tools currently used in the field were unprecedentedly revealed in the NMRI mouse model [55]. The metabolite changes recorded in this study directed further clinical research. The emphasis was placed on the path of glycolysis, amino acid metabolism as well as the intestinal microbiota, highlighting liver-specific human metabolic reactions, for example, the de novo synthesis and the secretion of primary bile acids (glycocholate, taurocholate, glycochenodeoxycholate, and taurochenodeoxycholate), as well as the degradation of ornithine. Gradually, the same metabolic pathways were exposed in other animal models of *Schistosoma* spp. infections [49,56,57] and confirmed later in human schistosomiasis [42,43,46], although with slight divergences. Such differences between studies in mouse models and humans are attributed to host factors, the intensity of infection which commonly differs between mouse models and humans, the unaccounted likelihood of coinfections in humans, and the dissimilar metabolic cascade leading to the morbidity profile. In addition, an interesting preliminary discovery was made by Wu and colleagues, who worked on progressive metabolic changes in schistosomiasis in Balb/C mice, identifying urinary 3-ureidopropionate (3-UP) as a potential biomarker for the early diagnosis of *Schistosoma* infection [58]. They observed elevated urinary 3-UP suggesting that *Schistosoma* infection resulted in reduced beta-ureidopropionase activity. However, in another study, Basant and colleagues also identified this metabolite as a potential biomarker for early malaria detection [59] showing that this metabolite fingerprint is not specific to schistosomiasis. Given the extensive co-endemicity of these 2 parasite infections, this may limit its use for future diagnostic development. However, the profile of this metabolite requires further investigations in human schistosomiasis, before being fully excluded.

Osakunor and colleagues tracked *S*. *haematobium* infection in populations of preschool-aged children living in schistosome endemic areas in Zimbabwe, demonstrating changes in metabolic profiles during the progression of natural infections over time [42]. Levels of creatinine, citrulline, sarcosine/N-methylglycine, and GABA were higher in boys when compared to girls, demonstrating a possible association with sexual dimorphism in changes of certain metabolites. They also showed that within 3 months of the first schistosome infection in young children, there were significant increases in AMP, ADP, 3-PG, and G6P, compared to uninfected children, and these increases were positively correlated with infection intensity. Analysis of metabolic pathways showed that the increases were related to energy (glycolysis) and purine metabolism which the authors attributed to schistosomiasis infection, despite the possible co-occurrence of other infections in the studied individuals.

In other studies, patients with schistosomiasis experienced dyslipidemia resulting in a reduction in total cholesterol, LDL, and triglycerides compared to healthy people [60,61]. Only metabologenomic models revealed the difference in the profile of lipid metabolism, suggesting the integration of genomic traits with metabolomic data can broaden and deepen the analysis. Such a metabolomics-based approach had been already successfully used in cardiology [62], metabolic syndrome [63], and disease diagnosis with high sensitivity and specificity for hepatitis C-infected patients [64]. This was achieved by comparing the candidate biomarker performance in several other causes of human liver diseases to rule out nonspecific candidates, e.g., alcoholic liver disease, nonalcoholic fatty liver disease (NAFLD), nonalcoholic fatty liver (NAFL), and nonalcoholic steatohepatitis (NASH) that might be co-occurring in the study settings. This approach could be tested in the human schistosomiasis model to attempt to distinguish people infected with *S*. *mansoni* from uninfected before and after PZQ chemotherapy and on the background of commonly co-occurring diseases such as malaria, hepatitis, and soil transmitted helminthiases.

Studies in Nigeria and Angola focusing on urogenital schistosomiasis have led to the discovery of numerous estrogen-like metabolites, as characteristic features of pathology, in the serum of infected individuals [65]. Metabolomic analysis of urine samples from these infected individuals revealed the presence of catechol estrogen quinones (CEQ) and CEQ-DNA adducts [65]. New metabolites derived from 8-oxo-7, 8-dihydro-2′-desoxyguanosine (8-oxodG) were also identified in the urine of 40 cases of urogenital schistosomiasis caused by *S*. *haematobium* [65]. More specifically, in the Nigerian participants affected by *S*. *haematobium*, depending on the severity of the infection, the metabolomic profiles obtained were associated with a reduction in the levels of sex steroids and high levels of several benzenoids, catechols, and lipids (including ganglioside, phosphatidylcholine, and phosphatidylethanolamine) in infected and diseased individuals when compared to uninfected and disease-free controls [43]. Together, these studies [43,65] have highlighted steroid trials as candidate biomarkers of urogenital schistosomiasis, which may represent a promising metabolomic avenue [66].

The picture has also been gradually drawn for metabolite markers of schistosomiasis associated pathologies, through various indirect evidences. Periportal fibrosis is one of the heaviest morbidities diagnosed in schistosomiasis parasites carriers. The liver is the most metabolically active tissue, and several models of metabolomic studies have been conducted to understand the development of liver fibrosis, although induced by noninfectious pathological conditions, and interesting metabolic pathways have been highlighted [67]. For example, in humans affected by NAFLD, fibrosis progression was associated with increased serum sulfate concentrations of 3 steroids: etiocholanolone (major metabolite of testosterone), dehydroepiandrosterone (precursor of androgens and estrogens), and 16α-hydroxy-dehydroepiandrosterone (precursor of estriol) [68]. The authors of this study concluded that the metabolites were ultimately associated with the progression of fibrosis in NAFLD [68]. However, it appears that they were not specific to this model since the key biomarker identified in these NAFLD studies, 16α-hydroxy-dehydroepiandrosterone sulfate, had also been reported in the serum of patients with breast cancer and endometrial cancer [69]. Such a lack of specificity for this biomarker in NAFLD-associated fibrosis highlights the limited likelihood of this biomarker providing a signature and sensitive measurement of fibrosis and not just cancer. Indeed, up to 20% of cancers are linked to inflammation-related chronic fibrosis [70]. Moreover, this candidate metabolite, 16α-hydroxy-dehydroepiandrosterone sulfate, is also an intermediate of estriol formation during pregnancy [71], making it clearly unsuitable for biomarking fibrosis specifically, and for a distinct disease condition in general. This further highlights the difficulty in identifying a clear disease-associated fibrosis-specific biomarker and further raises the need to test the performance of candidate biomarkers in several conditions to properly profile fibrosis and rule out ambiguity.

Another seminal attempt to biomark fibrosis was performed by Zhang and colleagues in 2006 [72]. They explored the use of targeted amino acid profiling to re-evaluate an association between liver fibrosis and fisher’s ratio, the molar ratio of branched amino acids to aromatic acids, achieving a fibrosis index based on amino acid concentration that could be applied to diagnose advanced liver fibrosis in chronic hepatitis C patients [72]. Other studies similarly established a strong correlation between morbidity and levels of free amino acids in the serum of hepatitis B-infected patients [73,74] altogether strengthening the clinical biomarking value of amino acid metabolism in the noninvasive monitoring of fibrosis, a pathognomonic feature of diseases including schistosomiasis. Therefore, Wang and colleagues went on to justify the use of amino acid metabolism as a diagnostic biomarker to classify morbidity according to the severity of schistosomiasis infection [55], highlighting the overall potential of metabolomics-based studies in revealing novel robust biomarkers of pathology during schistosomiasis.

**Fig 2:**
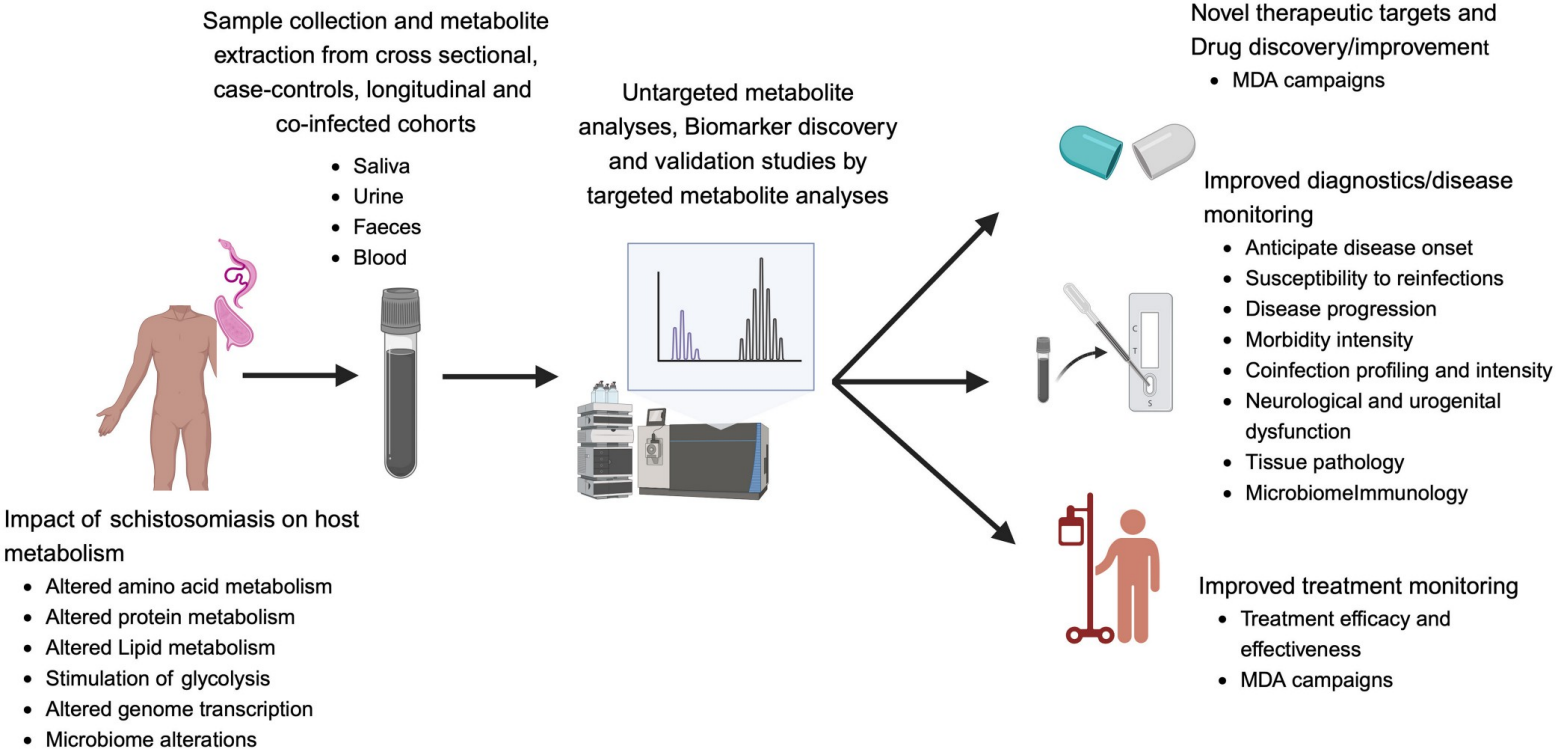
The influence of schistosomiasis on host metabolism and how it can be harnessed for diagnostic and therapeutic gains through metabolomics guided biomarker discovery. Created in Biorender.com.

Nevertheless, a striking limitation of such biomarking studies remained the lack of performance assessment of identified candidate biomarkers under conditions of defined coinfections, a setting that is the norm in schistosomiasis endemic areas [30,75,76]. A study was conducted by Gouveia and colleagues using 1H-NMR-based metabolomics models to diagnose periportal fibrosis caused by *S*. *mansoni* in patients either coinfected, or not, with chronic HBV or HCV infection [75]. A clear separation between coinfected and mono-infected (Schistosomiasis only) patients was reported using a supervised model built using partial least squares-discriminant analysis (PLS-DA) that succeeded in showing 100% precision predictive ability between mono-infected and coinfected individuals. This finding unprecedentedly supported a potential of metabolomic-based identification of candidate biomarkers as an alternative to conventional predictive methods for the diagnosis of periportal fibrosis caused by schistosomiasis even on a background of host multimorbidity. Building on these first observations from the metabolomics-based biomarking of the human host during *Schistosoma* infection and associated pathology, even on a complex background of coinfections, the addition of more metabolome signatures of schistosomiasis to the currently available diagnostic and morbidity monitoring tools would certainly strengthen our understanding of the host–parasite interactions, in addition to ameliorating our current control strategies through improved diagnostics (Fig 2).

Among the methods used in metabolomics involving mass spectrometry to identify new biomarkers, there are 3 types: (i) the untargeted metabolomics generally used to detect metabolites spike without prior knowledge; (ii) targeted metabolomics that analyze specific metabolites previously defined by the user; and (iii) a specialized form of targeted metabolomics combined with isotope flow also called fluxomic. The latter is achieved through the introduction of substrates labeled with heavy isotopes, which makes it possible to identify and quantify metabolites as they move through metabolic pathways. The heterogeneity of sensitivity of untargeted metabolomics is the main limitation compared to the 2 other targeted methods [77]. Thus, untargeted and targeted metabolomics can identify a plethora of metabolites acting differently in the metabolic pathways involved in schistosomiasis; however, none of them could tell us about their entangled conversion pathways deciphering the whole metabolic pathways along the progression of infection. By measuring the interconversion rates of metabolites, the stable isotope tracing technique will provide information not revealed by the untargeted conventional metabolomics [78], which could be a more powerful and complete approach to probe metabolic changes in pathological biological systems like in schistosomiasis infection. Thus, the application of serial circular studies on these different platforms of metabolomic investigations should be the key to helping elucidate previous inconclusive results of preclinical studies.

A fundamental requirement for a comprehensive assessment of the human host metabolomic variations of schistosomiasis infection and/or pathology is the refined phenotyping of patients from endemic areas, ensuring a wide range of age coverage (from preschool-aged children, school-aged children, to adolescent and adults), a mixed sex distribution (male and female), a diverse egg burden distribution (from low, moderate, and high burden infection candidates), and a comprehensive range of well-defined morbidities (from absent, mild, moderate to severe). This should enable a robust parallel between defined metabolome profiles and human host phenotypes during schistosomiasis.

Further analyses of a larger cohort of participants with schistosomiasis mono-infection or coinfections will also be essential to discovering relevant metabolites as biomarkers of infection and pathogenesis in *Schistosoma* spp. infections. Schistosomiasis infested sites are focal but rarely occur in isolation, and polyparasitism is the norm rather than the exception in endemic areas [75,76]. The robustness of host metabolome profiling should be unequivocally evaluated by the addition of coinfected individuals to the host metabolomics studies. Such an approach has been recently used for a canonical type-2 cytokine IL-33 and the biomarking potential for hepatosplenic schistosomiasis in a polyparasitic site of rural Cameroon [76]. This cytokine was negatively associated with schistosomiasis even in instances of host coinfection with hepatitis and malaria. Another key requirement for the identification of host metabolites that are pathognomonic of schistosomiasis infection and disease would be combining associative cross-sectional studies with follow-up longitudinal studies that would help validate the robustness of metabolite biomarkers identified. In the latter study design, the changes over time of the target metabolites alongside the infection and disease status of the host would be highly informative on the robustness of the candidate metabolite in real-life situations. Moreover, the use of cohorts undergoing treatment with praziquantel would help exclude non-robust candidate metabolites and uniquely reveal reliable metabolites that evolve in a timely manner with the host’s infection and disease status during schistosomiasis. Such attempts have already been undertaken in preclinical [79] as well as clinical settings [42], validating the power of such an approach of longitudinal follow up with treatment while also providing an exploitable list of candidate metabolite biomarkers during schistosomiasis. A consequent expectation would be the identification of metabolite biomarkers of infection acquisition or disease progression in individuals initially uninfected and disease-free upon long-term follow up in endemic areas. The use of such prognostic tools to accompany a test-and-treat strategy would be invaluable for the elimination of schistosomiasis whereby metabolite changes in finely clustered hosts could be robustly linked to the onset of schistosomiasis infection upon exposure and/or disease following infection. As such, the predictability of host metabolites for infectious disease acquisition and disease progression has been successfully tested in tuberculosis [80] demonstrating proof of concept. Critically, to ensure the informative value of such complex clinical sampling, phenotyping and metabolite assessment studies, sampling protocols are required, alongside strong clinical diagnostic skills and the accuracy of clinical and imaging techniques. Imaging for schistosomiasis pathology, as defined by the WHO, heavily relies on the local organometric and baseline values to conclude on abnormalities. As such, the generation of local referential of “normal” organometrics is critical as recently performed in rural Cameroon [81]. Only the use of such local referential can enable an accurate measurement of organ enlargement and thus disease grading, considering the dissimilar range of organometrics from one region to another [82]. Therefore, equipped with all these precautions, tools, and clinical settings, human metabolome profiling in the context of schistosomiasis and the wealth of robust metabolite biomarkers generated can then be further harnessed as bases to reliably evaluate the infectious status and severity of the disease process and its complications and further, perhaps enable species specific diagnosis as well as anticipate the risk of the individual being reinfected and/or developing infection-associated disease in the future.

The metabolomics profiling of schistosomiasis in humans can also be taken a step further than biomarker identification towards mechanisms of action [83]. As such, identified biomarkers can be utilized to detect how *Schistosoma* infection and/or associated disease impact biochemical pathways in the body. The key metabolites can be traced to metabolic pathways to understand which processes in the metabolic pathways are being affected in the body to produce the disease phenotype. Notably, however, the detection of altered biochemical pathways will require more advanced metabolomic techniques such as stable isotope tracing and integration of the data with other orthogonal datasets such as those resulting from other omics studies [83]. Tracing of the biochemical pathways would also require a firm understanding of the biochemical pathways and interactions to be able to tease out which parts are altered to drive the observed changes in the metabolome linked to the disease.

## Conclusions

Helminth infections, including schistosomiasis, cause changes in metabolism by altering their host metabolite production, as well as patterns of use, excretion, and absorption of metabolites. As current diagnostics and monitoring tools are insufficient for the detection and assessment of low-burden *Schistosoma* infections in humans and enabling the reliable monitoring of the severity of tissue pathology, more sensitive markers are urgently needed [27]. Consequently, achieving the 2030 target of schistosomiasis elimination as a public health problem requires a thorough understanding of the metabolic mechanisms involved in infection acquisition and the development of various morbidity traits associated with *Schistosoma* spp. infection. Thus, the present review suggests the incorporation of a metabolome fingerprint of *Schistosoma* infection and/or morbidity into available monitoring tools that might enable us to better understand host–parasite interactions and refine our current control strategies en route to eliminating schistosomiasis.

**Highlights**

- Low-burden infections of *Schistosoma* spp. are difficult to be reliably detected using the current microscopy-based diagnostic tools.
- *Schistosoma* spp. egg excretion counts and morbidity profiles may differ substantially, making it difficult to monitor schistosomiasis pathology with egg burden as the sole proxy.
- *Schistosoma* spp. infections and coinfections with other parasites, and co-morbidities further complicate host profiles.
- Metabolomics is an emerging approach that can provide, in a short time, clear information on metabolic changes in the host associated with infection and disease.
- Data on human hosts metabolomic profiles, with and without *Schistosoma* spp. infection, cross-sectionally and longitudinally, with and without treatment, with and without coinfections, are still limited but could provide a deeper understanding, improved biomarking, and ameliorated monitoring of *Schistosoma* spp. infection and associated morbidity.
- Biomarkers, identified by metabolomic profiling of human hosts, might lead to improved diagnostic tools and posttreatment monitoring of disease progression or regression.
