# Correction: Harnessing *Schistosoma*-associated metabolite changes in the human host to identify biomarkers of infection and morbidity: Where are we and what should we do next?

**DOI:** 10.1371/journal.pntd.0012480

**Published:** 2024-09-11

**Authors:** The PLOS Neglected Tropical Diseases Staff

The seventh author’s initials are indexed incorrectly in PubMed. The correct initials are: Nono JK.

The publisher apologizes for the error.
